# The effect of HIV infection on glycaemia and renal function in type 2 diabetic patients

**DOI:** 10.1371/journal.pone.0199946

**Published:** 2018-06-28

**Authors:** Siyabonga P. Khoza, Nigel J. Crowther, Sindeep Bhana

**Affiliations:** 1 Department of Chemical Pathology, National Health Laboratory Service and University of the Witwatersrand, Johannesburg, South Africa; 2 Department of Internal Medicine, Chris Hani Baragwanath Hospital, University of the Witwatersrand, Johannesburg, South Africa; Edith Cowan University, AUSTRALIA

## Abstract

**Background:**

Infection with, and treatment of HIV is associated with effects on glycaemia and renal function. The purpose of this study was therefore to compare glycaemic control and albuminuria in HIV-positive and HIV-negative type 2 diabetic patients.

**Materials and methods:**

Diabetic patients with and without HIV infection were recruited from a diabetic clinic at Chris Hani Baragwanath Hospital in Soweto, South Africa. Data was collected on weight, height, HbA1c, fasting glucose, urine albumin:creatinine ratio, HIV status, CD4 counts, viral load and concomitant therapies. Multivariable regression analysis was used to isolate the determinants of fasting glucose and HbA1c levels and risk factors for albuminuria.

**Results:**

Data were collected from 106 HIV-positive and 214 HIV-negative diabetics. All HIV infected subjects were receiving anti-retroviral therapy. The determinants of fasting glucose levels (log) were HIV infection (β = 0.04, p = 0.01) and use of anti-hypertensive agents (β = 0.07, p = 0.0006), whilst for HbA1c levels (log) they were HIV infection (β = -0.03, p = 0.03), BMI (β = 0.004, p = 0.0005), statin use (β = 0.04, p = 0.002) and glucose levels (β = 0.01, p<0.0005). In HIV-positive subjects, CD4 counts were negatively associated with glucose levels (β = -0.0002, p = 0.03). The risk factors for albuminuria were (odds ratio [95% CIs]) dyslipidaemia (1.94 [1.09, 3.44], p = 0.02) and HbA1c levels (1.24 [1.12, 1.38], p<0.0001).

**Discussion:**

These data suggest that glycaemic control is worse in type 2 diabetic subjects with HIV infection and that HbA1c underestimates glycaemia in these patients. Albuminuria was not associated with HIV-positivity. The negative relationship of CD4 counts with glucose levels may reflect viral removal and easing of the associated inflammatory response. It is possible that the association of statin and anti-hypertensive therapies with high HbA1c and glucose levels, respectively, is due to such therapies being given largely to subjects with poor glycaemic control.

## Introduction

Accessibility to highly active antiretroviral therapy (HAART) has dramatically improved the life expectancy of HIV-infected individuals, however studies have shown that HAART is associated with an increased prevalence of non-communicable diseases, including type 2 diabetes [1]. Thus, Brown et al. [2] observed that the prevalence of diabetes was up to four fold higher in HIV-infected men exposed to HAART compared to HIV-negative men. A recent meta-analysis confirms the strong association between the use of anti-retroviral therapy (ART) and increased risk for diabetes [3]. Furthermore, HIV-infected individuals have been found to have higher rates of metabolic syndrome, dyslipidaemia and lipodystrophy when compared to subjects without HIV infection [4].

The pathogenesis of type 2 diabetes in HIV-positive subjects involves several factors including the inflammatory response to the virus, co-infection such as hepatitis C virus and the use of HAART [4, 5]. Not only does HAART contribute to the aetiology of diabetes but studies have also shown that HIV-infected diabetic subjects receiving HAART appear to have poor glycaemic control [6]. However, only a limited number of such studies investigating the effect of HAART on glycaemia have been undertaken.

Kidney dysfunction, including both acute and chronic renal failure, is a known complication of HIV infection. The aetiology of HIV-associated acute kidney disease is thought to involve the virus itself, co-morbid diseases or infections and anti-retroviral therapy [7]. Thus, acute renal failure has been linked to the use of indinavir and tenofovir, and can progress to chronic kidney disease if not treated [8, 9]. Indinavir is associated with nephrolithiasis, papillary necrosis, and dysuria [8, 10] while tenofovir has been implicated in tubular toxicity and Fanconi syndrome [11]. With regard to chronic kidney disease (CKD), the prevalence of this disorder has been reported to be between 6.0–45% in HIV infected, ART-naïve subjects in sub-Saharan Africa [12]. The development of CKD in those infected with HIV is thought to be mediated by viral proteins, host genetic variants, and environmental factors [7].

The association of poorly controlled type 2 diabetes with kidney disease [13] suggests that HIV infection in diabetic subjects may further increase the risk of renal dysfunction. Very few studies have actually measured the prevalence of kidney disease in diabetic subjects with HIV however, 2 studies have shown that albuminuria was more prevalent in such subjects when compared to diabetic subjects who were HIV-negative [14,15].

It is therefore clear that HIV infection and its therapy may lead to both poor glucose control and an increased risk of kidney dysfunction in subjects with type 2 diabetes. This overlap of diseases and the resulting pathology is of significant concern in South Africa where both type 2 diabetes [16] and HIV infection [1] are common. Indeed, South Africa has the highest number of subjects living with HIV infection, with a national prevalence level of 12.2% [1]. In addition, a national survey has shown that the prevalence of type 2 diabetes in South Africa is 9.5% [16]. The aim of the present study was therefore to compare glycaemia and renal function (using albuminuria as a surrogate marker) in African type 2 diabetic patients with and without HIV infection, and to identify the determinants of fasting glucose and HbA1c levels and albuminuria in this population.

## Materials and methods

### Study population

The study was conducted at Chris Hani Baragwanath Academic Hospital diabetic clinic. The hospital is the referral centre for clinics in Soweto and surrounding communities. Consecutive type 2 diabetic patients of both genders were recruited over a period of 4 months. Patients above 30 years were included in the study while pregnant women and non-diabetic subjects were excluded from the study.

### Data and sample collection

Ethics approval was obtained for this study from the University of the Witwatersrand Ethics Committee (Human Research). All participants provided written informed consent before any study procedures were conducted. A data sheet was used to collect information from the patient and also from the patient’s file. During clinic visits fasting glucose, HbA1c and urine albumin:creatinine ratio were measured using point-of-care (POC) devices. The capillary blood samples for glucose and HbA1c measurement were obtained from the same puncture wound made using a sterile, disposable lancet, with the fingertip first being cleaned with an alcohol swab. A spot urine sample for the urine albumin:creatinine ratio measurement was collected into a sterile, disposable urine container from each participant during the clinic visit.

### Diabetes mellitus diagnosis

Diabetes was defined from self-report of ever being diagnosed with diabetes, and documentation that the patient was receiving anti-diabetic medication based on information in the patient files.

### HbA1c, urine albumin:creatinine ratio and glucose measurements

The HbA1c and urine albumin:creatinine measurements were performed using a Siemens DCA Vantage analyzer (Tarrytown, NY, USA), which is a cartridge-based analyzer. The DCA Vantage HbA1c assay method is based on a latex immunoagglutination inhibition methodology and it is certified by the National Glycohemoglobin Standardization Program (NGSP). The DCA Vantage has been assessed according to NGSP-certified HbA1c POC device guidelines and was found to have a 2.3% coefficient of variation (CV) at HbA1c levels of 5.26–5.34%, a CV of 2.5% over a HbA1c range of 6.10–6.18%, and a CV of 2.7% in the 8.01–8.09% range [17]. The assay has a reportable range from 2.5–14.0%.

The DCA Vantage albumin:creatinine assay is a quantitative method measuring albumin, and creatinine and reports a albumin:creatinine ratio. Urine albumin is linear between 5 to 300 mg/l and for creatinine linearity is between 1.3 to 44.2 mmol/l. The albumin:creatinine measurement covers the following range: 0.11 to 226 mg/mmol. Evaluation of the performance of this POC device in terms of measurement of urine albumin, creatinine and albumin:creatinine ratio against a laboratory-based method demonstrated excellent correlations (R^2^ = 0.989, 0.987 and 0.991, respectively) with total imprecision of < 8.7%, and an inter-and intra-day imprecision of < 2.9% [18]. Albuminuria was used as a surrogate marker for nephropathy, and was defined as a urine albumin:creatinine ratio of > 2.5 mg/mmol for men and >3.5 mg/mmol for women [19].

Fasting glucose levels were measured using a POC device, the Accu-Chek Active glucometer (Roche Diagnostics, Mannheim, Germany). It has been noted that this instrument has an imprecision of less than 5.5%, and based on a Clarke error grid analysis it demonstrated adequate clinical accuracy [20].

Quality control and calibration of the analysers were performed prior to measuring the patients’ samples according to the manufacturer’s recommendations.

### Other variables

Data obtained from the patients’ files included information on the use of ARVs, therapies for diabetes, dyslipidaemia and hypertension, CD4 counts and viral loads, duration of diabetes, duration since diagnosis of HIV, duration of ART and smoking. Hypertension was defined based on a physician’s diagnosis documented in the patient’s file and/or self-reported use of anti-hypertensive agents. Dyslipidaemia was defined as a self-reported history of use of lipid lowering agents and/or a diagnosis made by a physician and noted in the patient’s file. Weight and height measurements were recorded at the study visit.

### Statistical analyses

Data with a normal distribution is expressed as mean ± standard deviation in tables and text whilst data that is non-parametric is expressed as median (interquartile range). The χ^2^ test was used to compare categorical variables between groups whilst the Student non-paired t test was used for continuous variables following log transformation if required.

Multivariable linear regression models were developed to find the principal determinants of glucose and HbA1c levels and albumin:creatinine ratio (dependent variables). Univariate regression analyses were performed to determine the relationship of each study variable (independent variable) with each of the 3 dependent variables. Independent variables that correlated with the dependent variable at p≤0.20 were included in the multivariable model. Backward, stepwise removal of non-significant variables was then performed until a final model was obtained in which all remaining independent variables correlated with the dependent variable at p<0.10. Collinearity was assessed in all the initial multivariable models using the variance inflation factor (VIFs). Any variable with a VIF≥5 was removed from the model until all variables had VIF<5.

The sample size for this study was calculated based on the use of multivariable linear regression models for identifying the principal determinants of glucose and HbA1c levels in diabetic subjects with and without HIV infection. Assuming a maximum of 12 independent variables in the initial regression models, with a statistical power of 90%, a p-level of 0.05 and an effect size of 0.10, the estimated sample size was 230. We chose to use a sample size greater than this (N = 320) to ensure the study was fully powered to achieve its aims. Similar analyses were performed in sub-groups i.e. in those with and in those without HIV infection. These were secondary analyses and the sub-group sample sizes were not calculated to ensure that there was sufficient power to run these regression models. However, a *post hoc* power analysis demonstrated that in the sub-group with the smallest sample size i.e. HIV-positive subjects (n = 106), with 5 independent variables in the initial regression model that there was 85% power to identify a minimal effect size of 0.15 at p<0.05. All data from this study is included as supporting information (see S1 Data).

The Statistica software package was used for all statistical analyses (version 12, StatSoft, Tulsa, USA).

## Results

### Comparison of HIV-positive and HIV-negative subjects

The HIV-negative subjects were older (p<0.005), had a significantly higher BMI (p<0.005), and a longer duration of diabetes (p<0.05) than the HIV-positive subjects (Table 1). The higher HbA1c levels in the HIV-negative group (p<0.05) were found to be due to higher BMI and age as adjusting for either of these variables in an ANCOVA attenuated the p-value (p = 0.21 and p = 0.13, respectively). All subjects were receiving metformin in combination with insulin and/or sulphonylureas. All the HIV-positive patients were receiving ART with the majority using an anti-retroviral triple therapy of tenofovir, lamivudine, and efavirenz, with a very small number of subjects receiving stavudine (1 person) or zidovudine (also 1 person). Fewer of the HIV-positive subjects were being treated for hypertension (p<0.005) when compared to the HIV-negative subjects, but this relationship became non-significant after adjusting for age (p = 0.24). No difference in the prevalence of albuminuria was noted between the groups.

**Table 1:** Comparison of HIV-positive and HIV-negative subjects.

Variable	HIV-negative	HIV-positive
N	214	106
Females (%)	65.4	63.5
Age (years)	59.7 ± 9.83	53.9 ± 8.5**
BMI	33.1± 6.18	30.4 ± 5.04**
Smoker (%)	22.9	20.6
Diabetes duration (years)	13.3 ± 4.53	11.9 ± 5.04*
Diabetes therapy:		
Metformin (%)	100	100
Insulin (%)	91.1	87.8
Sulphonylurea (%)	31.8	32.7
Lipid therapy:		
Statins (%)	64.0	57.9
Fibrate (%)	3.27	1.90
Use of anti-hypertensives (%)	82.2	67.3**
Anti-retroviral therapy:		
Tenofovir (%)	-	86.0
Lamivudine (%)	-	87.8
Efavirenz (%)	-	87.8
Stavudine (%)	-	0.93
Zidovudine (%)	-	0.93
Duration of ART (years)	-	5.35 ± 4.19
CD4 count (cell/mm^3^)	-	633 ± 215
Viral load (copies/ml)	-	46.0 (0.50,111)
Viral load < 40 copies/ml (%)	-	48.8
HbA1c (%)	8.70 (7.20, 11.0)	8.20 (7.10, 10.0)*
Glucose (mmol/L)	8.20 (6.80, 10.0)	8.80 (6.90, 11.2)
Albuminuria (%)	31.3	35.2

Data expressed as mean ± SD, median (interquartile range) or %

*p<0.05

**p<0.005 vs. HIV-negative

### Multiple linear regression models for glucose and HbA1c

Stepwise, backward multivariable linear regression analysis demonstrated that HIV infection and the use of anti-hypertensive therapy were both independently associated with higher glucose levels (Table 2). Thus, HIV-positive subjects had glucose levels that were 4.40% higher than in HIV-negative subjects and subjects receiving anti-hypertensive therapy had glucose levels that were 6.90% higher than those not receiving such therapy (Table 2). With regards to HbA1c levels, HIV infection was negatively associated with this variable, whilst BMI, glucose and use of statins were positively associated. Thus, HIV-positive subjects had HbA1c levels that were 2.80% lower than those without HIV, a 1 unit increase in BMI was associated with a 0.30% increase in HbA1c, a 1 mmol/L increase in glucose levels was associated with a 1.20% increase in HbA1c, whilst subjects on statins had a 3.00% higher HbA1c level than those not taking statin therapy (Table 2).

**Table 2:** Multiple linear regression models for glucose and HbA1c.

Dependent variable	Independent variables with β-coefficient^a^ (p-value)	Adjusted R^2^ (p-value)
Log glucose (mmol/L)	HIV-positive: 0.044 (0.015) Use anti-HT: 0.069 (0.0006)	0.040 (0.0005)
Log HbA1c (%)	HIV-positive: -0.028 (0.027) BMI: 0.003 (0.007) Glucose: 0.012 (<0.0005) Use statins: 0.030 (0.002)	0.173 (<0.0005)

Anti-HT = anti-hypentensive therapy

^a^unstandardised β-coefficient

These data demonstrate that both glucose and HbA1c levels are determined by HIV status, and therefore we repeated the multivariable linear regression analyses of both these variables in HIV-negative and HIV-positive subjects. The results of these analyses are shown in Table 3. In terms of glucose levels, within the HIV-negative group only anti-hypertensive therapy was associated with glucose, with those using such therapy having a 6.30% higher glucose level than non-hypertensive subjects. In the HIV-positive group, BMI and CD4 counts were both associated with fasting glucose levels. Thus, a 1 unit increase in BMI was associated with a 0.60% increase in the glucose level, however this relationship just missed statistical significance (p = 0.084). A 1 unit increase in CD4 count was associated with a 0.02% fall in glucose concentration. With regard to HbA1c, in the HIV-negative group BMI, glucose and statin use were all correlated to this marker of glycaemia. Thus, a 1 unit increase in BMI was associated with a 0.30% rise in HbA1c, a 1 mmol/L increase in glucose was associated with a 1.40% rise in HbA1c and statin use was associated with a 4.00% higher HbA1c level. Within the HIV-positive group, gender, BMI, glucose and duration of HIV infection were all associated with the HbA1c level. Thus, females had a HbA1c level that was 4.00% lower than in males, a 1 unit increase in BMI was associated with a 0.40% rise in HbA1c, a 1 mmol/L increase in glucose was associated with a 0.90% rise in HbA1c and a 1 year higher duration of HIV infection was associated with a 0.30% increase in HbA1c. This last association just missed statistical significance (p = 0.074).

**Table 3:** Multiple linear regression models for glucose and HbA1c in HIV-negative and HIV-positive subjects.

Dependent variable	HIV status	Independent variables with β-coefficient^a^ (p-value)	Adjusted R^2^ (p-value)
Log glucose (mmol/L)	HIV-negative	Use anti-HT: 0.063 (0.012)	0.025 (0.012)
	HIV-positive	BMI: 0.006 (0.084) CD4 counts: -0.0002 (0.031)	0.050 (0.028)
Log HbA1c (%)	HIV-negative	BMI: 0.003 (0.022) Glucose: 0.014 (<0.0005) Use statins: 0.040 (0.009)	0.179 (<0.0005)
	HIV-positive	Gender: -0.040 (0.050) BMI: 0.004 (0.047) Glucose: 0.009 (0.0005) HIV duration: 0.003 (0.074)	0.185 (<0.0005)

Anti-HT = anti-hypentensive therapy

^a^unstandardised β-coefficient

gender was coded as females = 1 and males = 0

### Use of statins and anti-hypertensive agents

Due to the effects of statins on HbA1c and anti-hypertensive (HT) agents on glucose levels, these drugs were analysed in more detail. Patient files showed that simvastatin was the only statin type used in this population whereas 8 different types of anti-HT agents were used. The main agents used for treating hypertension (and their % use in subjects with hypertension) were: the diuretic, hydrochlorothiazide (38.6%); the ACE inhibitor, enalapril (45.3%); the calcium channel antagonist, adalat (25.6%). All these agents were used in combination with each other and with other anti-HT drugs. Due to the reported elevation of glucose levels by thiazide diuretics [21], these agents were compared against all other anti-HT drugs for their effects on glucose. Thus, in non-hypertensives, the median (interquartile range) fasting capillary glucose level was 7.10 (6.25, 9.30) mmol/L, in those using hydrochlorothiazide it was 8.65 (6.90, 11.0) mmol/L (p = 0.028 versus non-hypertensives) and in those using all other forms of anti-HT therapy it was 8.80 (7.10, 10.5) mmol/L (p = 0.033). Due to the large number of drug combination therapies (12) used in the non-thiazide group, we were not able to determine which individual therapies were the main contributors to the higher glucose level. No significant effects were observed for the anti-HT drugs on HbA1c levels.

### Multiple logistic regression models for albuminuria

The data in Table 4 show the results of backward, stepwise multivariable logistic regression analysis of albuminuria risk. The results demonstrate that gender, dyslipidaemia and HbA1c levels are all independent risk factors for albuminuria. Thus, the risk for albuminuria is 47.7% lower in females than males, and the risk of albuminuria is 94.2% higher is subjects with dyslipidaemia than in those with normal lipid levels. In addition, a 1 unit increase in HbA1c levels is associated with a 24.3% increase in the risk of albuminuria (Table 4).

**Table 4:** Multiple logistic regression model for albuminuria.

Dependent variable	Independent variables with odds ratio (95% CIs) and p-value
Albuminuria	Female gender: 0.523 (0.307, 0.893); 0.017 Dyslipidaemia: 1.942 (1.095, 3.444); 0.023 HbA1c: 1.243 (1.117, 1.383); <0.0005

## Discussion

The data from this study shows that HIV-positive diabetic subjects had poorer glycaemic control than HIV-negative subjects whilst HbA1c levels were lower in the former group after adjusting for possible confounding factors. Furthermore, within the HIV-positive group higher CD4 counts were associated with lower glucose levels. The use of anti-HT agents was found to be associated with poorer glycaemic control whilst the use of statins was linked to higher HbA1c levels. Kidney function, as assessed via albuminuria, was not different between the HIV-positive and HIV-negative diabetic groups. Albuminuria was found to be more prevalent in subjects with dyslipidaemia and in those with higher HbA1c levels.

This study has demonstrated that in diabetic subjects lower HbA1c levels are observed in HIV-positive compared to HIV-negative subjects, but higher glucose levels in the former compared to the latter group. This has also been observed in other studies [22–24], only 1 of which was undertaken in diabetic subjects [23]. These data demonstrate that HbA1c measurement in HIV-positive diabetic subjects underestimates the level of glycaemia. Whilst HbA1c is an excellent tool for monitoring glycaemic control, there are several factors that can affect its level by influencing the lifespan of red blood cells. These factors include iron/vitamin B12 deficiency, chronic renal failure, splenectomy, splenomegaly, increased haemolysis, cirrhosis and drugs such as ribavirin and dapsone [25]. It has been suggested that in HIV-positive subjects, the lower HbA1c levels may be as a result of a faster turnover of red blood cells due to increased haemolysis [22]. The haemolysis may be due to the use of ART, and a number of studies have shown that the discrepancy between glucose and HbA1c levels is related to such therapy [22–24]. Furthermore, a meta-analysis of studies conducted in sub-Saharan Africa has shown that ART-use is associated with lower HbA1c measurements [26]. It is therefore advised that fasting glucose measurements be used for the diagnosis of diabetes in HIV-positive subjects [27], and this is in line with the American Diabetes Association guidelines which discourage the use of HbA1c as a diagnostic or a glycaemia monitoring tool in HIV infected subjects [28].

The current study demonstrated that glucose levels were higher in diabetic subjects with HIV infection when compared to those who were not infected. A recent South African study also demonstrated poor glycaemic control in diabetic patients living with HIV [6], using data collected from patient files. A study from the USA showed that serum glucose levels were higher in diabetic, HIV infected subjects compared to those without HIV but the difference missed statistical significance (p = 0.09) [23]. Furthermore, a longitudinal investigation demonstrated that newly diagnosed diabetic subjects with HIV, when initiated onto anti-diabetic therapy showed a poorer improvement in glycaemic control than a group of HIV-negative subjects [29]. However, a further study showed no difference in fasting glucose levels between female diabetic subjects with or without HIV infection [30], whilst a study from Malawi demonstrated lower fasting glucose levels in HIV-positive compared to HIV-negative diabetic subjects [14]. It can therefore be seen that there are only a few studies that have analysed the effect of HIV infection on glycaemic control in type 2 diabetic subjects. The majority of these studies suggest that HIV infection is associated with poorer glycaemic control. It has been suggested that this is due to the effects of ART [29], and it is interesting to note that in the study showing lower glucose levels in HIV-positive compared to HIV-negative diabetic subjects, only 16.9% (11 of 65) were receiving ART [14].

We were able to show that within the diabetic subjects with HIV infection, the CD4 count correlated negatively with glucose levels. A similar association has been observed in a previous study, which was conducted in non-diabetic subjects [31]. These data suggest that improved immune system functionality may enhance glycaemic control, which may be mediated by a reduction of the viral load and/or attenuation of the inflammatory response to viral infection. It is known that inflammation may play an important role in the aetiology of type 2 diabetes [32]. Conversely, the use of ART can give rise to immune reconstitution inflammatory syndrome (IRIS) in a sub-population of HIV infected subjects [33]. The effect of IRIS on the development of type 2 diabetes in subjects with HIV infection has not been investigated, but should be the focus of future studies. The present study also demonstrated that in HIV-positive subjects a longer duration of HIV infection was associated with higher HbA1c levels. However, this association was very weak.

The use of anti-HT agents was associated with higher glucose levels whilst statin use was associated with higher HbA1c levels. Eriksson et al found that higher glucose levels were associated with hydrochlorothiazide therapy compared to placebo in hypertensive subjects [34], whilst a meta-analysis has shown a significant glycaemic effect of both thiazide diuretics and non-selective beta blockers in subjects with diabetes [21]. The present study also demonstrated an association of both thiazide and non-thiazide therapy with higher glucose levels. With regard to statin therapy, Liew et al reported an increase in HbA1c levels in patients receiving simvastatin regardless of the presence of diabetes [35]. This parallels the current study where simvastatin use was associated with higher HbA1c levels. In addition, a meta-analysis has demonstrated a modest increase of HbA1c in diabetic patients receiving statin therapy [36]. However, it is possible that the relationship observed of anti-hypertensive and statin therapy with glucose and HbA1c levels, respectively, are simply due to the provision of these drugs to subjects who have the worst glycaemic control. In order to clarify the relationship of these therapies with glycaemia future longitudinal studies would be required.

The current study demonstrated that albuminuria was not associated with HIV infection. However, it must be noted that only 2 previous studies have investigated this relationship [14,15], both of which showed a higher prevalence of albuminuria in diabetic subjects that were HIV-positive. One of these studies concluded that albuminuria was related to the use of abacavir [15], whilst the other study involved HIV-positive subjects of whom only 16.9% were receiving ART [14]. Within the current study, all HIV-positive subjects were receiving ART but none were using abacavir. These data suggest a complex interaction between HIV infection, its therapy and kidney function, with lack of ART and use of particular anti-retroviral agents both being related to albuminuria. We hypothesize that full ART coverage in the HIV-positive subjects may protect them from HIV-related kidney dysfunction and may explain the similar level of albuminuria within the 2 patient groups. The present study showed that albuminuria was associated with the presence of dyslipidaemia and with raised HbA1c levels. Similarly, a study from Korea demonstrated that dyslipidaemia was associated with an increased risk of albuminuria in pre-diabetic female subjects [37]. Furthermore, it is known that kidney disease can lead to dyslipidemia which in turn can contribute to the development of diabetic nephropathy [38].

Within the current study, albuminuria was used as a surrogate measure of renal function. This choice was based on its ease of measurement using POC technology and the fact that the urine albumin:creatinine ratio is a commonly used indicator of diabetic nephropathy [39]. Furthermore, a number of large meta-analyses have shown that albuminuria predicts end-stage renal disease irrespective of gender [40] or the presence or absence of hypertension [41] or diabetes [42].

The principle anti-retroviral drugs used by the HIV-positive participants in this study were efavirenz, tenofovir and lamivudine. These drugs are recommended by the Southern African HIV Clinicians Society as the preferred first line therapy for HIV infection and are the most readily available anti-retroviral agents within the public healthcare system of South Africa [43], and the majority of low- and middle-income countries [44]. Stavudine and zidovudine were used by very few of the participants. These drugs are still available within South Africa but are only recommended for use in cases where multiple other drugs are contraindicated, and in the case of stavudine, use is recommended for no longer than 3 months [43]. Within South Africa, protease inhibitors are recommended as second line therapy for HIV infection, whilst integrase inhibitors are recommended for third line therapy [43].

Limitations of this study include the use of a single urine albumin-creatinine ratio as a surrogate marker of renal function. In addition, the use of POC devices for measuring glucose, HbA1c and the albumin-creatinine ratio may be considered a drawback, however such devices are increasingly being used in both clinical and epidemiological studies and show good agreement with laboratory-based methodologies [17,18,20]. A number of variables in this study were based on data taken from patient files and this may limit the conclusions that can be drawn when using such information. All HIV-positive subjects were receiving ART and therefore we were not able to discern whether any differences between subjects with and without infection were due to the virus or its therapy. The sub-group analyses were not powered to the same extent as those performed in the full cohort and therefore the results of these regression models should be interpreted with some caution. Despite this, significant associations were still identified in the sub-group regression analyses. Lastly, this study was conducted in a single out-patient clinic in an urban setting and our findings may therefore not be applicable to all HIV-positive diabetics within South Africa. The strengths of this study include the assessment of a broad range of appropriate variables and that it was sufficiently powered to detect differences in glucose and HbA1c levels between the 2 study groups. Furthermore, this is the first cross-sectional study to specifically assess glycaemic control and kidney function in sub-Saharan African diabetic subjects with HIV infection and receiving HAART.

In conclusion, these data show that HIV-positive diabetic subjects receiving ART have higher fasting glucose levels than HIV-negative diabetics. In addition, statin and anti-HT therapy were associated with poorer glycaemic control, the origins of which are uncertain. Furthermore, fasting glucose levels were negatively associated with CD4 counts, a relationship that requires further investigation. The prevalence of albuminuria did not differ between HIV-negative and HIV-positive diabetic patients, a finding that was hypothesized to be due to full ART coverage in the latter group. Due to the high prevalence of both HIV infection and type 2 diabetes in sub-Saharan Africa it is important that future studies further investigate the clinical consequences of the overlap of these 2 epidemics and put in place programs to help ameliorate these effects.

## Supporting information

S1 Data Dataset Khoza et al. This file contains all data from the paper. (XLS)
